# Classifying Included and Excluded Exons in Exon Skipping Event Using Histone Modifications

**DOI:** 10.3389/fgene.2018.00433

**Published:** 2018-10-01

**Authors:** Wei Chen, Pengmian Feng, Hui Ding, Hao Lin

**Affiliations:** ^1^Center for Genomics and Computational Biology, School of Life Science, North China University of Science and Technology, Tangshan, China; ^2^Innovative Institute of Chinese Medicine and Pharmacy, Chengdu University of Traditional Chinese Medicine, Chengdu, China; ^3^School of Public Health, North China University of Science and Technology, Tangshan, China; ^4^Key Laboratory for Neuro-Information of Ministry of Education, Center of Bioinformatics and Center for Information in Biomedicine, School of Life Science and Technology, University of Electronic Science and Technology of China, Chengdu, China

**Keywords:** alternative splicing, exon skipping, histone methylation, histone acetylation, random forest

## Abstract

Alternative splicing (AS) not only ensures the diversity of gene expression products, but also closely correlated with genetic diseases. Therefore, knowledge about regulatory mechanisms of AS will provide useful clues for understanding its biological functions. In the current study, a random forest based method was developed to classify included and excluded exons in exon skipping event. In this method, the samples in the dataset were encoded by using optimal histone modification features which were optimized by using the Maximum Relevance Maximum Distance (MRMD) feature selection technique. The proposed method obtained an accuracy of 72.91% in 10-fold cross validation test and outperformed existing methods. Meanwhile, we also systematically analyzed the distribution of histone modifications between included and excluded exons and discovered their preference in both kinds of exons, which might provide insights into researches on the regulatory mechanisms of alternative splicing.

## Introduction

RNA splicing is a process that eliminates introns from the precursor messenger RNA (pre-mRNA) so that exons can be linked together, which is an essential step of gene expression (Tilgner et al., 2012). In some cases, RNA splicing can create a range of unique proteins by orchestrating exons of the same pre-mRNA in different modes (Black, 2003). This phenomenon is known as alternative splicing. Among the numerous modes of alternative splicing, exon skipping is the most common one, in which a particular exon may be included in mRNAs under some conditions and omitted from the mRNA in others (Black, 2003).

It has been demonstrated that ~95% of human genes undergo alternative splicing (Wang et al., 2008a). The multiple transcript variants of alternative splicing from a single gene often have different biological functions. However, our knowledge about the regulatory mechanism of alternative splicing is far from satisfactory.

In the past decades, a series of researches have been carried out in order to reveal the mechanisms of alternative splicing, and demonstrated that alternative splicing is regulated not only on the genome level but also on the epigenome level (Fox-Walsh and Fu, 2010). On the genome level, there are exonic and intronic splicing enhancers (ESEs and ISEs) and silencers (ESSs and ISSs), which are sequence motifs that can be recognized and bound by proteins (Wang and Burge, 2008; Barash et al., 2010). Although the information on genome level can explain some of the splicing events, it is not sufficient for cell type specific and stage type specific RNA splicing (Wang et al., 2008a).

Recent researches have demonstrated that histone modifications from the epigenome level also participate in medicating RNA splicing. For example, Luco et al. have demonstrated that the alternative splicing of the FGFR2 (Fibroblast growth factor receptor 2) gene is regulated by H3K36me3 (Luco et al., 2010). Zhou et al. found that the exon inclusion event of human Fibronectin (FN1) gene is medicated by H3K9me2 and H3K27me3 (Zhou et al., 2014). Shindo et al. found that combinatorial effect of histone modifications also contribute to alternative splicing patterns among different cell lines (Shindo et al., 2013). These results hint us that finding the splicing code from histone modifications will provide new insights into RNA splicing regulatory mechanisms.

Accordingly, several computational methods have been proposed to classify included and excluded exons in exon skipping event based on histone modifications. In 2012, Enroth et al. developed a rule-based model and obtained an accuracy of 72% (Enroth et al., 2012). Later on, Chen et al. proposed a quadratic discriminant (QD) function based method and obtained an accuracy of 68.5% (Chen et al., 2014). More recently, by integrating features of genomic sequences and histone modifications, Xu et al. proposed a deep learning approach to predict splicing patterns (Xu et al., 2017). These works promote the research progress on revealing RNA splicing regulatory mechanisms. However, the performance of these methods remains unsatisfactory.

In the current study, we proposed a new method to classify included and excluded exons in exon skipping event. The Maximum Relevance Maximum Distance (MRMD) feature selection technique was used to winnow out the optimal histone modification features. By using the histone modification information, the Random Forest (RF) was performed to establish the prediction model. Results of 10-fold cross validation test demonstrate that the proposed method is reliable.

## Materials and methods

### Dataset

The dataset used to train and test the predictive model was constructed by Enroth et al. (Enroth et al., 2012). According to the gene expression data of CD4^+^ T cell, Enroth et al. obtained 13,374 “included” and 11,587 “excluded” exons from the exon skipping event of the human genome (Enroth et al., 2012). These exons are all 50 bp long with flanking introns longer than 360 bp, and none of them overlap to each other. Enroth et al. further mapped the 20 kinds of histone acetylation (Barski et al., 2007) and 18 kinds of histone methylation (Wang et al., 2008b) to those exons and their closest 180 bp of flanking intronic regions. By doing so, they obtained the histone modification signals and represented them by binary attributes, namely present (noted by “1”) and absent (noted by “0”) over the three regions (preceding, on and succeeding the exons). After removing exons with no histone acetylation or methylation modification present, a benchmark dataset containing 12,692 “included” exons and 11,165 “excluded” exons with histone acetylation and methylation information was obtained.

### Sample formulation

By using the binary attributes of 20 kinds of histone acetylation and 18 kinds of histone methylation (Supplementary Table S1), the samples in the dataset can be represented by a 114-dimensional vector given by

(1) $$R=\;{\left[\Phi_{1},\;\Phi_{2},\;\Phi_{3},\cdots \;\Phi_{i},\cdots ,\;\Phi_{114}\right]}^{T}$$

where **T** is the transpose operator. The values for the vector component Φ_*i*_ can be “1” (indicating the presence of histone modification) or “0” (indicating the absence of histone modification). Φ_1_, Φ_2_, *and Φ*_3_ indicate the presence or absence of H3K27me3 on, preceding and succeeding exons, respectively; Φ_4_, Φ_5_, *and Φ*_6_ indicate the presence or absence information for H3K4me2, and so forth. More details can be found in Supplementary Table S1. The encoded samples by using histone modification information are available at https://github.com/chenweiimu/splicing.

### Feature selection

If the exons are represented by a vector of 114 dimensions, it may bring out the following three unfavorable problems (Feng et al., 2013): (1) including redundant or irrelevant information; (2) leading to over-fitting problems and reducing the generalization capacity of the model; (3) increasing the computational time. In order to alleviate irrelevant features, a series of effective feature selection techniques have been proposed, such as analysis of variance (Lin and Ding, 2011; Lin et al., 2015), Minimal Redundancy Maximal Relevance (Peng et al., 2005; Chen et al., 2014), and Diffusion Maps (Coifman et al., 2005).

In this study, the Maximum Relevance Maximum Distance (MRMD) approach was employed to select the optimal features, which has been widely used in the realm of bioinformatics since proposed in 2016 (Zou et al., 2016). As indicated by Zou et al. (2016), the major concern of MRMD is searching a kind of features ranking metric which contains two aspects: one is the relevance between sub feature set and target class, and the other is redundancy of sub feature set. The more details about MRMD can be found in Zou et al.'s work 2016.

### Random forest

Random forest (RF) is an ensemble of a large number of decision trees (Breiman, 2001). Each tree in the ensemble is trained on a subset of training instances that are randomly selected from the given training set. Instead of using all the features, a random subset of features is selected, further randomizing the tree. The prediction results of RF are based on the ensemble of those decision trees and each tree gives a classification result. Finally, the RF classifier selects the prediction result that has the largest number of votes from the classification results. Owing to its advantages in dealing with high-dimensional data, RF has been used in various areas of bioinformatics (Ferrat et al., 2018; Manavalan et al., 2018; Wang et al., 2018).

### Cross validation

In statistical prediction, three cross-validation methods, namely independent dataset test, sub-sampling (or n-fold cross-validation) test and jackknife test, are often used to evaluate the anticipated success rate of a predictor. Among the three cross-validation methods, the jackknife test is deemed the least arbitrary and most objective one (Chen et al., 2015, 2018; Feng et al., 2018). However, to reduce the computational time, the 10-fold cross validation test was used to evaluate the performance of the proposed method. For 10-fold cross-validation, the training dataset is randomly partitioned into ten training subsets, and nine subsets were used for training and the remaining one was used for testing. This process was repeated ten times in such a way to ensure that each set is utilized once for testing the model that was trained on the other nine.

### Performance evaluation

The performance of the proposed method was evaluated by using the following four metrics, namely sensitivity (*Sn*), specificity (*Sp*), Accuracy (*Acc*), and the Mathew's correlation coefficient (*MCC*), which are expressed as (Chen et al., 2017; Lin et al., 2017; Jia et al., 2018; Zeng et al., 2018)

(2) $$\{\begin{array}{l} Sn=\frac{TP}{TP+FN}\times 100\%\\ {}[2mm]Sp=\frac{TN}{TN+FP}\times 100\%\\ {}[2mm]Acc=\frac{TP+TN}{TP+FN+TN+FP}\times 100\%\\ {}[2mm]MCC=\frac{\left(TP\times TN\right)-\left(FP\times FN\right)}{\sqrt{\left(TP+FN\right)\times \left(TP+FP\right)\times \left(TN+FN\right)\times \left(TN+FP\right)}} \end{array}$$

where *TP, TN, FP*, and *FN* represent true positive, true negative, false positive, and false negative, respectively.

## Results and discussion

### Performance evaluation

By encoding the included and excluded exons in the dataset using the histone modification, each of the sample was represented by a 114-dimensional vector (Equation 1) used as the input vector of RF to build a computational model. By examining the performance of the model via the 10-fold cross-validation test, we obtained an accuracy of 63.49%, which is still far from our satisfaction. In order to improve the performance of the proposed model, it is necessary to choose the optimal number of features to build a robust and efficient predictive model.

**Figure 1:**
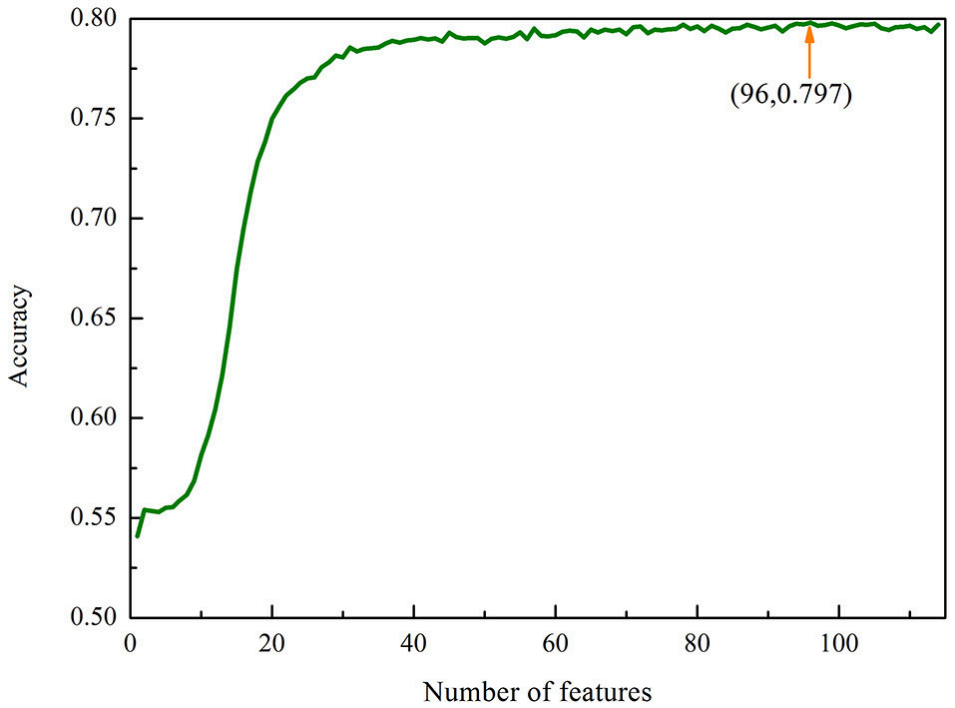
The IFS curve for classifying “included” and “excluded” exons in the exon skipping event. An IFS peak of 79.79% was obtained when using the optimal 96 features to perform predictions.

We therefore used the MRMD together with the Incremental Feature Selection (IFS) strategy to build the optimal feature subsets. We ranked the 114 features using the MRMD algorithm. The 114 ranked features were then added one by one from lower to higher rank. This procedure was repeated 114 times, and for each time a RF model was built. Their performances were investigated by using the 5-fold cross-validation test. The most optimal features can be obtained when the accuracy reaches its maximum. The IFS was used to determine the optimal number of features. The corresponding IFS curve was plotted in Figure 1. Accuracy reaches its maximum of 79.79% when the top ranked 96 features were used to encode the samples. Therefore, a computational model was built based on these 96 optimal features. In this case, the proposed model obtained an accuracy of 72.91% with the sensitivity of 67.03% and specificity of 79.65% in 10-fold cross-validation test.

### Comparative analysis among different classifiers

To further demonstrate the power of the proposed method for classifying the ‘included' and “excluded” exons, we compared its performance with that of other classifiers, such as BayseNet, Naïve Bayes, J48 Tree and Support Vector Machine (SVM). All these classifiers were tested on the benchmark dataset and implemented in WEKA (Frank et al., 2004) with the default settings. Their 10-fold cross-validation test results based on the 96 optimal features were reported in Table 1. As indicated in Table 1, the four metrics as defined in Equation. 2 for the current method are all higher than those of BayseNet and SVM. Although Naïve Bayes and SVM yielded higher sensitivity, their specificity, accuracy, and MCC are significantly lower than that of the current method.

**Table 1:** Performance metrics of different classifiers for classifying included and excluded exons.

**Method**	**Sn (%)**	**Sp (%)**	**Acc (%)**	**MCC**
BayseNet	66.84	55.02	61.33	0.22
Naïve Bayes	68.00	53.58	61.25	0.22
J48 Tree	61.06	53.20	57.38	0.14
SVM	67.82	59.72	64.05	0.27
Random Forest	67.03	79.65	72.91	0.46

In addition, a comparison was also made between the current method and the method in our previous work (Chen et al., 2014), where a QD function based method was proposed to classify the “included” and “excluded” exons. Since both methods are trained and tested based on the same dataset, we directly compared the 10 fold cross-validation test results of the current method with that listed in previous work (Chen et al., 2014). As indicated in Table 2, the accuracy achieved by the current method is over 4% higher than existing method, indicating that the current method is superior to our previous method for classifying the “included” and “excluded” exons.

**Table 2:** A comparison of the current method with existing method for classifying included and excluded exons.

**Method**	**Sn (%)**	**Sp (%)**	**Acc (%)**	**MCC**
Chen et al's method^a^	68.90	66.70	68.50	–
Current method	67.03	79.65	72.91	0.46

a *(Chen et al., 2014)*.

### Features analysis

To provide an overall view of the optimal features for classifying the “included” and “excluded” exons, we compared their frequency distributions in both kinds of exons using the *z*-test (Table 3). As we can see from Table 3, among the 96 optimal features, 29 features significantly prefer to the included exons, while 52 features significantly prefer to the exclude exons. More interestingly, 61 of the 81 features that differently distributed in “included” and “excluded” exons are from the proceeding or succeeding regions of the exons. This result indicates that the major regulatory epigenetic factors of exon skipping event located in the surrounding regions of the exons.

**Table 3:** The 96 optimal features and their bias to exon inclusion or exclusion case^a^.

**Feature**	**Bias**	**Feature**	**Bias**	**Feature**	**Bias**
H3R2me1.succ	I	H3K36me1.succ	E	H4K5ac	E
H3R2me1.prec	I	H3K18ac.prec	I	H4K20me1.prec	–
H4K8ac.succ	I	H4K91ac.prec	I	H4K20me1.succ	E
H4K12ac.prec	E	H3K23ac.succ	I	H2AK5ac	E
H4K8ac.prec	E	H3K36me1.prec	E	H3K23ac	I
H4K12ac.succ	–	H3K23ac.prec	E	H3K79me1.succ	–
H3K36me3.succ	E	H4R3me2.succ	I	H3K36me1	–
H3K9ac.succ	E	H2BK120ac.prec	I	H3K79me1.prec	E
H3K14ac.prec	E	H4R3me2.prec	I	H2BK20ac	E
H3K27me3.succ	E	H3K9me1.prec	E	H2BK12ac	E
H3K27me3.prec	I	H2BK120ac.succ	E	H4K16ac	–
H3K9ac.prec	–	H3K9me1.succ	I	H3K4ac	E
H3K14ac.succ	–	H3R2me2.prec	I	H2BK5me1	E
H2AK5ac.prec	E	H2AK9ac.succ	I	H3K18ac	I
H2AK5ac.succ	E	H3R2me2.succ	E	H3K9me2	I
H4K5ac.succ	E	H2AK9ac.prec	E	H4R3me2	I
H4K5ac.prec	I	H3K27ac.prec	E	H3K4me1.prec	E
H2BK20ac.succ	–	H3K27ac.succ	E	H3K4me1.succ	E
H2BK20ac.prec	–	H3K36me3	E	H2AK9ac	E
H4K16ac.prec	E	H3K9me2.succ	I	H3K4me2.prec	E
H4K16ac.succ	E	H3R2me1	I	H3K4me2.succ	I
H3K36me3.prec	E	H4K8ac	I	H4K91ac	–
H3K4ac.succ	I	H2BK5ac.prec	I	H3K9me1	–
H3K4ac.prec	E	H3K14ac	–	H3R2me2	–
H2BK12ac.prec	E	H3K9me2.prec	–	H2BK120ac	E
H2BK12ac.succ	I	H4K12ac	I	H3K79me3.succ	E
H2BK5me1.succ	E	H2BK5ac.succ	E	H3K9me3.succ	E
H2BK5me1.prec	E	H3K27me3	E	H3K9me3.prec	E
H3K27me2.succ	I	H3K27me1.succ	I	H3K79me3.prec	E
H3K27me2.prec	E	H3K9ac	E	H3K36ac.succ	I
H4K91ac.succ	E	H3K27me2	E	H3K27me1	E
H3K18ac.succ	E	H3K27me1.prec	E	H3K27ac	E

a *The bias of the 96 optimal features to exon inclusion or exclusion case were analyzed using hypothesis test of sample frequency. “I” indicates that he features that significantly (p < 0.01) bias to exon inclusion case, while “E” indicates bias significantly (p < 0.01) bias to exon exclusion case*.

**Figure 2:**
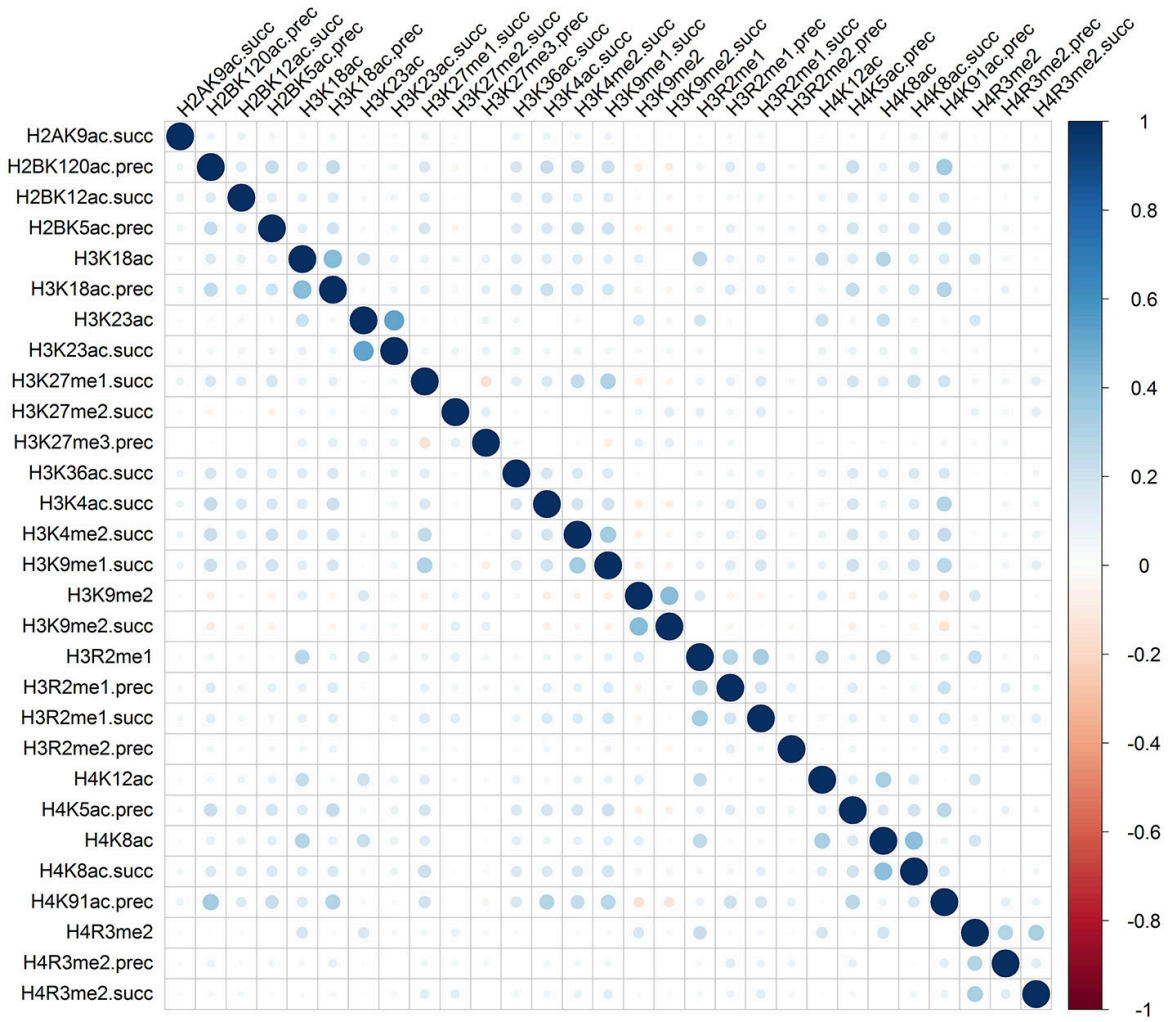
Correlation matrix of histone modifications for the exon inclusion case of exon skipping event.

**Figure 3:**
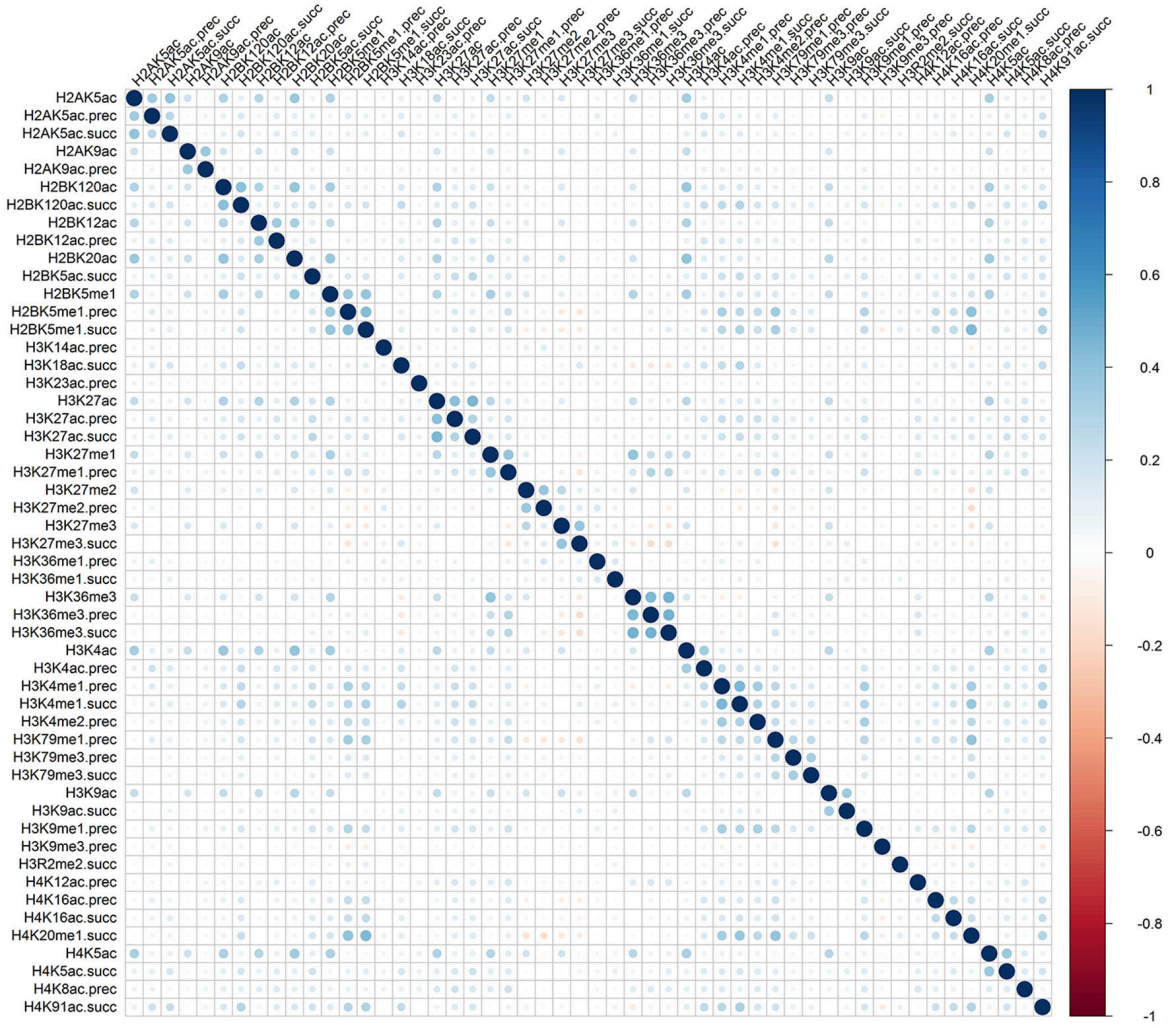
Correlation matrix of histone modifications for the exon exclusion case of exon skipping event.

Rather than medicated by a single type of histone modification, recent researches have demonstrated that RNA splicing can be regulated by a combination of different types of histone modifications (Shindo et al., 2013). To detect whether the cooperation or competition of histone modifications exists in the exon skipping event process, we calculated the Pearson correlation coefficient of the 81 optimal features. The correlation matrix for “included” and “excluded” exons were plotted in Figures 2, 3, respectively. As indicated in these figures, significant positive and negative correlations could be observed among different kinds of histone modifications. For example, in the “included” exon case, H3K18ac is positively correlated with H3K23ac, H4K8ac and H4K12ac, while H4K91ac is negatively correlated with H3K91me2. In the “excluded” exon case, H2AK5ac is positively correlated with H2BK5me1, H2BK12ac, H2BK20ac, H4K5ac, and H3K4ac; the negative correlations are observed between H3K79me1 with H3K27me2, H3K27me3, and H3K6me1. These results prove that the histone modification cooperation and competition indeed exist in the process of RNA splicing.

## Conclusion

As one of the key processes of gene expression, besides regulated by ESEs, ISEs, ESSs, ISSs, and other trans-elements, RNA splicing is also regulated by epigenetic factors. In this paper, we presented a new computational method to classify the “included” and “excluded” exons in exon skipping events based on histone modifications. The samples in the dataset were encoded using optimal histone modification information obtained by feature selection technique and then used as the input of RF. The predictive results derived by the 10-fold cross validation test demonstrated that the proposed approach can achieve better performance than existing approaches.

To provide an intuitive view of the histone modifications that contribute to the predictions, we systematically analyzed their distributions in “included” and “excluded” exons. The non-random distribution of histone modifications (Table 3) and their positive or negative correlation profiles (Figures 2, 3) suggest that exon skipping is regulated by the combination of different types of histone modifications. Further experimental investigations are required to reveal how these histone modifications are associated with splicing.

In the future work, we will do our best to develop a much more smart method to classify “included” and “excluded” exons by integrating information from both the genome and epigenome levels.

## Author contributions

WC and HL conceived and designed the experiments. PF and HD performed the experiments. HL and WC wrote the paper. All authors read and approved the final manuscript.

### Conflict of interest statement

The authors declare that the research was conducted in the absence of any commercial or financial relationships that could be construed as a potential conflict of interest.
