# Supplementary material for: Classifying Included and Excluded Exons in Exon Skipping Event Using Histone Modifications
Source: Front Genet. 2018 Oct 1;9:433. doi: 10.3389/fgene.2018.00433 (PMC6174203; doi:10.3389/fgene.2018.00433)
Supplement: Supplementary file 1 [file Table_1.DOCX]

***Supplementary Material***

**Classifying included and excluded exons in exon skipping event using histone modifications**

**Wei Chen^1,2^*, Pengmian Feng^3^, Hui Ding^4^, Hao Lin^4^***

1. Center for Genomics and Computational Biology, School of Life Science, North China University of Science and Technology, Tangshan, Tangshan 063000, China;
2. Innovative Institute of Chinese Medicine and Pharmacy, Chengdu University of Traditional Chinese Medicine, Chengdu 611730, China;
3. School of Public Health, North China University of Science and Technology, Tangshan, 063000, China;
4. Key Laboratory for Neuro-Information of Ministry of Education, Center of Bioinformatics and Center for Information in Biomedicine, School of Life Science and Technology, University of Electronic Science and Technology of China, Chengdu 610054, China;

**Supplementary Table S1.** 38 kinds of histone modifications and their corresponding vector elements in Eq. 2 of the main text.

| Feature | Element | Feature | Element | Feature | Element |
| --- | --- | --- | --- | --- | --- |
| H3K27me3 | Φ_1_ | H3K79me1_succ | Φ_39_ | H2AK9ac_prec | Φ_77_ |
| H3K27me3_prec | Φ_2_ | H3K9me2 | Φ_40_ | H2AK9ac_succ | Φ_78_ |
| H3K27me3_succ | Φ_3_ | H3K9me2_prec | Φ_41_ | H2BK5ac | Φ_79_ |
| H3K4me2 | Φ_4_ | H3K9me2_succ | Φ_42_ | H2BK5ac_prec | Φ_80_ |
| H3K4me2_prec | Φ_5_ | H4K20me1 | Φ_43_ | H2BK5ac_succ | Φ_81_ |
| H3K4me2_succ | Φ_6_ | H4K20me1_prec | Φ_44_ | H3K27ac | Φ_82_ |
| H3K79me3 | Φ_7_ | H4K20me1_succ | Φ_45_ | H3K27ac_prec | Φ_83_ |
| H3K79me3_prec | Φ_8_ | H3K27me2 | Φ_46_ | H3K27ac_succ | Φ_84_ |
| H3K79me3_succ | Φ_9_ | H3K27me2_prec | Φ_47_ | H4K12ac | Φ_85_ |
| H3R2me1 | Φ_10_ | H3K27me2_succ | Φ_48_ | H4K12ac_prec | Φ_86_ |
| H3R2me1_prec | Φ_11_ | H3K4me1 | Φ_49_ | H4K12ac_succ | Φ_87_ |
| H3R2me1_succ | Φ_12_ | H3K4me1_prec | Φ_50_ | H4K91ac | Φ_88_ |
| H4R3me2 | Φ_13_ | H3K4me1_succ | Φ_51_ | H4K91ac_prec | Φ_89_ |
| H4R3me2_prec | Φ_14_ | H3K79me2 | Φ_52_ | H4K91ac_succ | Φ_90_ |
| H4R3me2_succ | Φ_15_ | H3K79me2_prec | Φ_53_ | H2BK120ac | Φ_91_ |
| H2BK5me1 | Φ_16_ | H3K79me2_succ | Φ_54_ | H2BK120ac_prec | Φ_92_ |
| H2BK5me1_prec | Φ_17_ | H3K9me3 | Φ_55_ | H2BK120ac_succ | Φ_93_ |
| H2BK5me1_succ | Φ_18_ | H3K9me3_prec | Φ_56_ | H3K14ac | Φ_94_ |
| H3K36me1 | Φ_19_ | H3K9me3_succ | Φ_57_ | H3K14ac_prec | Φ_95_ |
| H3K36me1_prec | Φ_20_ | H4K20me3 | Φ_58_ | H3K14ac_succ | Φ_96_ |
| H3K36me1_succ | Φ_21_ | H4K20me3_prec | Φ_59_ | H3K36ac | Φ_97_ |
| H3K4me3 | Φ_22_ | H4K20me3_succ | Φ_60_ | H3K36ac_prec | Φ_98_ |
| H3K4me3_prec | Φ_23_ | H2AK5ac | Φ_61_ | H3K36ac_succ | Φ_99_ |
| H3K4me3_succ | Φ_24_ | H2AK5ac_prec | Φ_62_ | H4K16ac | Φ_100_ |
| H3K9me1 | Φ_25_ | H2AK5ac_succ | Φ_63_ | H4K16ac_prec | Φ_101_ |
| H3K9me1_prec | Φ_26_ | H2BK20ac | Φ_64_ | H4K16ac_succ | Φ_102_ |
| H3K9me1_succ | Φ_27_ | H2BK20ac_prec | Φ_65_ | H2BK12ac | Φ_103_ |
| H3R2me2 | Φ_28_ | H2BK20ac_succ | Φ_66_ | H2BK12ac_prec | Φ_104_ |
| H3R2me2_prec | Φ_29_ | H3K23ac | Φ_67_ | H2BK12ac_succ | Φ_105_ |
| H3R2me2_succ | Φ_30_ | H3K23ac_prec | Φ_68_ | H3K18ac | Φ_106_ |
| H3K27me1 | Φ_31_ | H3K23ac_succ | Φ_69_ | H3K18ac_prec | Φ_107_ |
| H3K27me1_prec | Φ_32_ | H3K9ac | Φ_70_ | H3K18ac_succ | Φ_108_ |
| H3K27me1_succ | Φ_33_ | H3K9ac_prec | Φ_71_ | H3K4ac | Φ_109_ |
| H3K36me3 | Φ_34_ | H3K9ac_succ | Φ_72_ | H3K4ac_prec | Φ_110_ |
| H3K36me3_prec | Φ_35_ | H4K8ac | Φ_73_ | H3K4ac_succ | Φ_111_ |
| H3K36me3_succ | Φ_36_ | H4K8ac_prec | Φ_74_ | H4K5ac | Φ_112_ |
| H3K79me1 | Φ_37_ | H4K8ac_succ | Φ_75_ | H4K5ac_prec | Φ_113_ |
| H3K79me1_prec | Φ_38_ | H2AK9ac | Φ_76_ | H4K5ac_succ | Φ_114_ |
